# Supplementary material for: Patterns of Intron Gain and Loss in Fungi
Source: PLoS Biol. 2004 Nov 30;2(12):e422. doi: 10.1371/journal.pbio.0020422 (PMC532390; doi:10.1371/journal.pbio.0020422)
Supplement: Table S1 — Also available at http://genes.mit.edu/NielsenEtAl/. (4.3 MB ZIP). [file pbio.0020422.st001.zip › NielsenEtAl/html/330.html]

AN2877.1.NCU08578.1.MG05718.1.FG02771.1


```
 CLUSTAL W (1.82) Multiple Sequence Alignments - Introns Inserted


Sequence 1: NCU08578.1	390 aa
Sequence 2: MG05718.1	418 aa
Sequence 3: FG02771.1	413 aa
Sequence 4: AN2877.1	415 aa
Alignment Length: 426 aa
Number Identitical Residues: 187 aa
Alignment Score (without introns) 9285


MG05718.1 	MADSTPTQNPQATG-SSWTAFLK0SIASFNGDLSSLTAPPFILSGTSLTEFSSYWCEHPT
NCU08578.1	MDSAQQNSSAAAGGGSSWGAFLK0SIASFNGDLASLTAPPFILSGTSLTEFSSYWCEHPP
FG02771.1 	-MSSAANPSAQANS-SSWTAFLK0SIASFNGDLSSLTAPPFILSSQSLTEFSAYWATHPP
AN2877.1  	MSSKDTASGLPAQNKSTWSSFLK0SIASFNGDLSSLTAPPFILSGTSLTEYSAYWAEHPA
          	  .     .  * . *:* :*** *********:**********. ****:*:**. **.

MG05718.1 	LFAAPAKESDPEKRALLVLKWFISTLKQQYASRSEQYGNEKKPLNPFLGELFLGKWEDEA
NCU08578.1	VFAAPAKESDPAKRALLVLKWFLTTLKQQYASRSEQYGNEKKPLNPFLGELFLGKWEDEA
FG02771.1 	VLTAPAAESDPAKRAMLVLKWFLSTLKHQYASRSEQFGNEKKPLNPFLGELFLGTWSDEA
AN2877.1  	LFVAPAREADPEKRALAVLKWFLSTLRQQYCSRSEKLGSEKKPLNPFLGELFLGKWDSDA
          	::.*** *:** ***: *****::**::**.****: *.***************.*..:*

MG05718.1 	--GTTELISEQVN2HHPPATAYSITNVPTGVR~LEGYNAQKATFSKTINVKQIGHAVLTV
NCU08578.1	--GVTELISEQVS2HHPPATAYNITNLPTGVR~LEGYNAQKASFSKTINIKQIGHAVLTV
FG02771.1 	--GETTLVSEQVS~HHPPATAYCIRNNKTGVE~LEGYNAQKATFKSTIIVKQIGHAVLRV
AN2877.1  	NIGETTLISEQVS2HHPPATAYAIRNEKHGVQ0LQGYNAQKASFSSTIYIKQIGHALYTL
          	. * * *:****. ******** * *   **. *:*******:*..** :******:  :

MG05718.1 	PVPGN----ASKKETFLITLPSLHIEGLIFGSPFVELDGATYITSSSGYTSKIDYSGKGW
NCU08578.1	PSP-T----SDQPDTFLITLPSLHIEGLIFGSPFIELDGASYITSSTGYTAKIDYSGKGW
FG02771.1 	PLA------SGEIESYLITLPGLHIEGLIFGAPFVELDGSSHITSSSGFTCKIDYSGKGW
AN2877.1  	TPPGADKNDPAQQEKYVITLPALHVESLIYGNPFMELEKSTYIVSSTGYISKISYSGKGW
          	. .. ..... : :.::****.**:*.**:* **:**: :::*.**:*: .**.******

MG05718.1 	LSGKKNSVTAVMYPTGK-EKEVLYNVTGQWTTTFEIHAGPAKQNKSSNLVDTYTASEHPS
NCU08578.1	LSGKKNSFVASLYPTGK-EKEVLYNVSGQWTKSFEIHSGAAKHNSKSTLVDSWDPEKQHH
FG02771.1 	LSGKKNTIVASVYPTGK-EKDVLYNVTGQWNKSFEVYSGPAKSNSKSTLVSTYDAVSTAQ
AN2877.1  	VSGKKNTFTASLYKESEGEKKPLYTVDGQWSNTFTIKDARIKKD-----VETVSVQDLQT
          	:*****:..* :*  .:.**. **.* ***..:* :  .  * :     *.:    .   

MG05718.1 	SKLIVAPIEQQHPLESRRAWAKVAAAIAKTELETVHVEKSKIEQAQRDLRAKEKAEGRMW
NCU08578.1	TPLTVAPIEKQHPLESRRAWSKVANAILKGDLEAVGQEKSRIEVAQREARAREKAEG---
FG02771.1 	TELHVAPIDQQHPLESRRAWSRVAEAIQKGDMDTTGAEKGKIEHAQRNMRTVERSEGRTW
AN2877.1  	TPLTLAPIEEQDLYESRRAWSDVAANIETGNMDAVSAAKGKIENAQRELRKVEKAEGREW
          	: * :***::*.  ******: **  * . ::::.   *.:** ***: *  *::**   

MG05718.1 	QRRYFSIRTDAPDEVLTTLGPVVGLA~THGDSDRTGGLWRFDEAKAEKAKAEPKLTPEQE
NCU08578.1	-------------------------P1EHGDADKTGGLWRFDATKAEKEREQPQLSDEEA
FG02771.1 	DRQYFTAVE-GQDPVLQQLGNVVGVP~ADGEADKTGGLWRFDKAKAD-ARVAKQLTQDDI
AN2877.1  	ERRFFERVDENQDQQFLELARKAG-L~TSLESDKTGGVWRFSPEKAANAEPPYHKVGGEG
          	.        .  .      .   .      ::*:***:***.  ** . .   :    : 

MG05718.1 	AKIAKECLGQ
NCU08578.1	SKIAKELLGQ
FG02771.1 	AQMEKELLGW
AN2877.1  	LGVSN-----
          	  : :
```
